# Obstetric Trauma due to Abuse and Preventable Events: A Commentary on Definition, Classification and Qualitative Research

**DOI:** 10.1111/1471-0528.18316

**Published:** 2025-07-24

**Authors:** Ronald F. Lamont, Laura K. Fraser, Carmen Amezcua‐Prieto, Jan Stener Jørgensen

**Affiliations:** ^1^ Research Unit of Gynaecology and Obstetrics, Department of Gynaecology and Obstetrics, Institute of Clinical Research University of Southern Denmark Odense C Denmark; ^2^ Department of Obstetrics and Gynecology Sygehus Sønderjylland Aabenraa Denmark; ^3^ Department of Preventive Medicine and Public Health University of Granada Granada Spain; ^4^ Consortium for Biomedical Research in Epidemiology and Public Health (CIBERESP) Madrid Spain; ^5^ Instituto de Investigación Biosanitaria de Granada (ibs.GRANADA) Granada Spain

**Keywords:** delivery: birth trauma, health services research, maternity services

## 1 Introduction

In recent months, two important documents were published that addressed ‘birth trauma’ and ‘obstetric violence’. The first of these, in May 2024, was the UK All Party Parliamentary Group (APPG) in Westminster that marked the launch of the first ever inquiry/report in the United Kingdom on BirthTrauma (https://www.theoclarke.org.uk/sites/www.theoclarke.org.uk/files/202405/Birth%20Trauma%20Inquiry%20Report%20for%20Publication_May13_2024.pdf). The second document, published in January 2025, was a well‐conducted Systematic Review (SR) of obstetric violence in high‐income countries comprising a mixture of qualitative and quantitative studies [1].

### 1.1 Comparative Methodologies and Populations of the APPG Report and Systematic Review

The APPG inquiry received more than 1300 submissions from mothers and fathers who had experienced traumatic birth in the United Kingdom, as well as nearly 100 submissions from maternity healthcare professionals (HCPs). The APPG took invited, written evidence from parents and HCPs about their experience of traumatic birth. Witnesses were asked to provide their evidence as free‐text submissions of up to 1500 words. The inquiry also carried out seven oral evidence sessions, each with a different theme. In contrast, the SR included 25 studies comprising 65 343 women from 15 high‐income countries outside the United Kingdom (Australia, Chile, France, Germany, Ireland, Israel, Italy, Netherlands, Poland, Portugal, Saudi Arabia, Spain [4], Sweden, Switzerland [3] and the USA [6]). The SR included quantitative, qualitative and mixed studies.

The APPG inquiry's objectives were to: (i) identify common features in maternity care that contribute to birth trauma; (ii) highlight examples of good practice; (iii) consider the impact of birth trauma on women's relationships (bonding, future pregnancies, returning to work); (iv) assess current postnatal services to treat women's physical and mental health problems; (v) develop parameters for understanding the possible economic cost of birth trauma with a view to future research; and (vi) influence government policy by identifying where maternity care could be improved to minimise birth trauma. These objectives matched many of the findings of the SR on obstetric violence, which is closely linked with birth trauma, and both found that there was disrespect and abuse in obstetric care. The key message of both was that: (i) physical, verbal and psychosocial obstetric violence was highly prevalent in high‐income countries; and (ii) coercion and lack of information or consent were some of the domains most frequently reported by women across all study types.

Unfortunately, due to the concomitant timing of publication of these two documents, it was not possible to combine their impact and point out their similarities and/or differences. Furthermore, the SR exposed the evidence as being sparse and difficult to identify due to the lack of Medical Subject Heading (MeSH) terms and key words to conduct a literature search. In the literature, the terms obstetric violence, trauma, mistreatment and abuse were used inconsistently and interchangeably. An indication of the novel nature of this subject of research is that none of the 25 studies included in the SR was published before 2019, and none of the data was collected before 2016. With no existing MeSH terms or synonyms for obstetric violence, and even using six respected databases, it was difficult to find relevant studies that satisfied the sensitivity and specificity of the search [1].

A positive experience of childbirth can empower women. However, preventable violence that causes traumatic childbirth may have long‐term consequences on the pregnant woman, such as post‐traumatic stress syndrome [2], delay in return to work and fear of future childbirth that may contribute to increased rates of caesarean section. In addition, attendant HCPs also suffer following traumatic birth [3, 4], the effects of which contribute to HCP burnout that in turn may also adversely affect patient care [5]. Violence in obstetric care may also have negative, long‐term consequences on the partner who witnesses these events. HCPs and partners can be considered second victims in these situations. Accordingly, we are grateful for this opportunity to propose: (i) an internationally accepted definition of violence in obstetric care; (ii) a comprehensive classification of this area of research; and (iii) an explanation of the differences between quantitative and qualitative research, both of which, but particularly the latter, would be appropriate research techniques for this topic. This would hopefully provide the perfect opportunity to bring together the many different aspects of damage to women's health in and around pregnancy.

## 2 Standardisation: Definitions and Classification Frameworks

### 2.1 Definitions

That this topic is a novel subject for research is manifest by the confusion over nomenclature.

We are not aware of any internationally accepted definition for obstetric violence/trauma/mistreatment etc. As a definition, the APPG cited ‘a woman's experience of interactions and/or events directly related to childbirth that caused overwhelming distressing emotions and reactions, leading to short‐ and/or long‐term negative impacts on a woman's health and wellbeing’ [6]. We feel that this is too restrictive and should be further expanded with respect to ‘the what’ (nature), ‘the who’ (subject), ‘the when’ (timing) and ‘the how’ (method). In our investigation of this topic, we encountered several different terms to describe this subject (Table 1). In addition, none of the terminologies used were sufficiently comprehensive to include: (i) different stages of pregnancy (antenatal, intrapartum or post‐partum); (ii) women, their partners and attendant HCPs; and (iii) whether the adverse events were physical, verbal, psychosocial or by omission. Furthermore, in proposing a definition, it is difficult to avoid the use of words such as ‘violence’, ‘abuse’ or ‘trauma’, because these may refer to cause or effect, and may depend on a balance between ‘susceptibility’ and ‘exposure’ wherein the response to a particular insult (in its broadest sense) may vary in degree according to the weighting of the adverse effect per se. Accordingly, we have chosen the umbrella term ‘violence in obstetric care’ and would like to propose the following definition: ‘An event, either intentional or unintentional, that occurs during the antenatal, intrapartum or postnatal period. These events can be physical, verbal, psychosocial or negligent in nature and are caused by the actions of the HCP and experienced by the birthing woman’. We have chosen to use the term ‘violence in obstetric care’ to highlight that the violence can take place in all stages of obstetric care and is not only limited to violence from maternity staff. This nomenclature is also broad enough to include hospital‐based births and home births.

**TABLE 1:** Some different terms that have been used to describe adverse events due to obstetric abuse causing trauma.

‘Violation of woman's rights’
‘Disrespect and abuse’
‘Obstetric mistreatment’
‘Abuse in healthcare’
‘Dehumanised care’
‘Mistreatment of women’
‘Detrimental care’
‘Violence in maternal care’

‘Obstetric violence’ has been the most prominent nomenclature within this area of research. It has also been polarising, as HCPs may not feel comfortable with the thought that they could be implicit in violence. Violence can however be considered as a violation of integrity, where ‘integrity’ refers to a state of wholeness or being intact, that is, something that is not broken. Accordingly, an act of violence can be understood as something that damages the status quo [7]. When considered in this context, the term ‘violence’ may be an appropriate descriptor for the phenomenon (see Table 2) to elicit damage to the integrity of the birthing woman. This definition is victim‐orientated, as it permits the subjectivity of the woman's experience.

**TABLE 2:** Classification of the nature and examples of violence in obstetric care, either intentional or unintentional, that occur during the antenatal, intrapartum or postnatal period. Violence in obstetric care can be further categorised into the following subsections: physical, verbal, psychosocial or negligent in nature. The table is not exhaustive, but a sample of the responses from women and their partners who have experienced violence in obstetric care. For completeness, the reader is referred to the full text of both publications on which this commentary is based (APPG and SR).

	Nature of abuse	Examples
A	Physical	Procedures carried out without informed consent, for example, episiotomy or vaginal examination Procedures carried out without medical indication Hitting, pushing or slapping Kristeller manoeuvre (manual fundal pressure during second stage of labour)
B	Verbal	Humiliation Raised voice in communication Bullying, scolding or mocking Making threats
C	Psychosocial	Denial of partner presence at birth Use of coercion to gain consent Violations of privacy Failure of HCP to listen, lack of compassion or poor rapport Racism Stigmatisation or discrimination based on sexual orientation, ethnic group or religion
D	Omission or negligence	Lack of access to interpreter services Lack of access to analgesia Lack of informed consent Abandonment, absence or non‐attendance of HCP upon request for help Failure to exercise duty of candour/cover up of errors Lack of help with breast feeding problems Lack of information or access to mental health support Failure to meet professional standards of care

Abbreviation: HCP, healthcare professional.

### 2.2 Classifications

In our suggested classification framework (Table 2), we did not include self‐harm, albeit that recent confidential enquiries into maternal deaths have consistently found that, excluding COVID‐19 deaths, mental health‐related causes continue to account for a large proportion (34%) of maternal deaths. In addition, we did not include IPV (intimate partner violence) because this type of violence/abuse is out of the control of HCPs. Nevertheless, this information on self‐harm and IPV may not be volunteered by pregnant women and so should be part of direct questioning by the HCP at booking. Equally, we have not included pregnancy in areas of conflict, which no doubt affects pregnant women disadvantageously, but is also mainly out of the control of hospitals and their HCPs.

Our suggested classification of the nature of violence in obstetric care is listed in Table 2. These examples are not exhaustive but a comprehensive sample of feedback from pregnant and puerperal women and their partners regarding their experiences from both the reports under scrutiny (APPG and SR). It is notable that virtually all the examples listed as the lifetime experiences of women and their partners are common to both reports. A few experiences were noticeably more commonly raised, such as lack of consent, access to analgesia, and poor communication between patients, their partners and HCPs. The reader is referred to the Ockenden Report (2022), which stresses the importance of actively listening to and engaging with families as well as providing accurate and evidence‐based information to allow women to participate equally in all decision‐making processes (https://www.gov.uk/government/publications/final‐report‐of‐the‐ockenden‐review/ockenden‐review‐summary‐of‐findings‐conclusions‐and‐essential‐actions).

The most recent MBRRACE‐UK report (2024) concluded that communication with recent migrant women with language difficulties should be provided with information about how to access maternity services in a variety of formats, settings and languages. The provision of professional interpreter services can enhance hospital care for linguistically diverse patients by improving patient–provider communication [8].

Consent was also a common issue and is particularly important as it now has medicolegal implications. Although the case of Montgomery v Lanarkshire established that patients should be fully informed about all the risks associated with the management options [9], this is still not happening in clinical practice. Rather than advising the patient on which management the doctor thinks would be best for the patient, the patient must now be informed of all the management options that are available, together with the relative risks associated with each and the patient should have the choice of therapy. The same legal change on informed consent occurred in Australia many years ago [10]. Currently in the United Kingdom, duty of candour also has medicolegal implications, and this also appeared in many patients' experiences. The creation of professional and statutory duties of candour through the UK GMC (General Medical Council) has formalised the requirement for clinicians to be honest with patients and families when treatment/interventions have gone wrong. The concept of candour, the role of apologies and the evidence about compliance is well covered elsewhere [11]. Another common experience among women during the intrapartum period was lack of access to analgesia, and for post‐partum women, being left alone for long periods of time, particularly being left in soiled bedclothes and suffering a delay before perineal repair could be performed following instrumental or spontaneous delivery. A significant number of women in the APPG report complained of poor support in the post‐partum period, particularly with respect to access to psychosocial therapy. Accordingly, it is of interest to note that one of the APPG report's pre‐inquiry objectives was to ‘assess current postnatal services to treat women's physical and mental health problems’. Finally, the use of the Kristeller manoeuvre, which involves manual fundal pressure during 2nd stage to expedite delivery, was listed as a mistreatment in five of 25 papers (20%) included in the SR. However, although sometimes used in UK midwifery practice and associated with significant discomfort, the procedure is rarely referred to by this terminology so did not appear in the APPG report. Increasingly, the Kristeller manoeuvre has medicolegal implications [12].

## 3 The Value of Qualitative Research in Trauma Studies

Most medical researchers use quantitative research methods, yet qualitative research for ‘lived experiences’ is an enormously valuable tool. Many investigators are familiar with quantitative studies but are fearful of qualitative studies because they equate them with poorly thought‐out questionnaire studies that lack sample size, validation and reproducibility [13]. That is not the case with well‐conducted qualitative studies in which rigorous qualitative methods can capture nuances often missed in quantitative datasets.

### 3.1 Lived Experiences

Lived experience refers to the direct and personal knowledge and understanding obtained from an individual who has lived through a particular situation or event. It covers the individual's feelings and perceptions in the context of their own human experience. This contrasts with relying upon reading about or being informed about an individual's experience by someone else. Lived experiences can be invaluable in planning policy and improving practices in the development of more effective solutions and can help researchers understand individuals' perspectives and experiences in greater depth.

### 3.2 Phenomenology

Phenomenological research is often used in qualitative research. In phenomenology, lived experiences are the main object of study, but the goal is not to understand individuals' lived experiences as facts, but to understand the meaning of such experiences. Accordingly, in phenomenology, lived experience is not reflective about an experience while living through it, but is recollective, with a given experience being reflected on after it has ended.

A good example of a qualitative study using a phenomenological methodology approach was to examine the experience of women with a pre‐pregnancy BMI of > 30 kg/m^2^, in their encounters with HCPs during pregnancy. Face‐to‐face, qualitative, in‐depth interviews with 16 pregnant women were carried out in the woman's own home. The participants' lived experiences (a term also used in the APPG report) of their encounters with HCPs were recorded verbatim, transcribed and analysed using a software phenomenological approach. Two main themes were identified: (i) an accusatorial response from HCPs and (ii) lack of advice and helpful information on how being obese and pregnant might affect the women's health and that of her child. The conclusion was that obese pregnant women may experience prejudice from HCPs. These women felt they were treated with a lack of respect, an accusatorial response and the feeling that information which could have been helpful was not forthcoming [14]. Another good example of qualitative research in pregnancy involving lived experience is recommended [15].

## 4 Conclusion

We commend the readers to read in full both reports referred to in this commentary. In common, both reports only included data from high‐income countries. We also commend the reader to the comprehensive Australian, New South Wales Senate Birth Trauma inquiry report, published around the same time as the APPG report (https://www.parliament.nsw.gov.au/lcdocs/inquiries/2965/FINAL%20Birth%20Trauma%20Report%20‐%2029%20April%202024.pdf).

In addition, in March of this year, with the publication of Law no. 33/2025, Portugal became the first country in the European Union to legally recognise obstetric violence. The Law established concrete measures for prevention, awareness and oversight of the problem. However, the law also showed significant limitations with respect to interpersonal dynamics, institutional and structural dimensions and informed consent. In addition, the legislative process excluded meaningful participation from HCPs, initiating a backlash from medical associations such as the Portuguese Medical Council and Nursing Council, both of which have requested the law's revocation. See: https://respectfulcare.eu/obstetric‐violence‐recognised‐in‐portuguese‐national‐law/.

In this commentary, we have tried to add structure and order to the eclectic nomenclature used to describe common, yet avoidable adverse outcomes that have short‐ and long‐term physical and psychosocial effects on pregnant women, their partners and their attendant HCPs.

To name a problem is to recognise its existence and acknowledge that, not only does the problem exist, but that we must do something about it, like raising its profile and taking action to fix it. We hope that through our comments on definition and classification, we have contributed to the first step of this process to allow the establishment of best practice and improvements to minimise such problems in the future.

**Summary**

Violence in obstetric care is a novel area of research, because of which nomenclature is confusing and requires an internationally acceptable definition. We have suggested the term “violence in obstetric care”, with the following definition: “An event, either intentional or unintentional, that occurs during the antenatal, intrapartum or postnatal period. These events can be physical, verbal, psychosocial or negligent in nature and are caused by the actions of the HCP and experienced by the birthing woman.” We have also suggested a classification which we feel encompasses the nature, the subject, the timing and the method of this abuse/damage‐based trauma. Qualitative research is a very useful approach to elicit and evaluate “lived experiences”. We suggest MeSH terms and keywords for future searches such as : Abandonment, Abuse, Anaesthesia, Analgesia, Antenatal, Breast Feeding Support, Bullying, Candour, Care Below Acceptable Standards, Coercion, Compassion, Consent, Cover‐up, Damage, Dehumanisation, Disrespect, Detriment, Healthcare Professionals, Humiliation, Interpreter Services, Intrapartum, Mental Health Support, Mistreatment, Negligence, Obstetric, Omission, Partner, Physical, Postnatal, Pregnancy, Preventable, Privacy, Psychosocial, Racism, Rights, Standards of Care, Stigmatisation, Trauma, Unintentional, Violation, Verbal, Violence, Violence in Obstetric Care, Women.

## Author Contributions

Conceptualisation: R.F.L. and J.S.J. Original draft preparation: R.F.L. All authors were involved in the review, revision and editing of the manuscript and have read and agreed to the published version of the manuscript.

## Conflicts of Interest

The authors declare no conflicts of interest.

## Data Availability

Data sharing is not applicable to this article as no datasets were generated or analysed during the current study.
